# Immunogenicity of eGFP-Marked Recombinant *Lactobacillus casei* against Transmissible Gastroenteritis Virus and Porcine Epidemic Diarrhea Virus

**DOI:** 10.3390/v9100274

**Published:** 2017-09-25

**Authors:** Meiling Yu, Li Wang, Sunting Ma, Xiaona Wang, Yusai Wang, Ya Xiao, Yanping Jiang, Xinyuan Qiao, Lijie Tang, Yigang Xu, Yijing Li

**Affiliations:** 1 College of Veterinary Medicine, Northeast Agricultural University, Harbin 150030, China; yu19890130@126.com (M.Y.); wanglicau@163.com (L.W.); masunting@163.com (S.M.); xiaonawang0319@163.com (X.W.); bggfgg163@163.com (Y.W.); 18845756200@163.com (Y.Xi.); jiangyanping2017@126.com (Y.J.); qiaoxinyuan@126.com (X.Q.); tanglijie@neau.edu.cn (L.T.); 2 Heilongjiang Key Laboratory for Animal Disease Control and Pharmaceutical Development, Harbin 150030, China

**Keywords:** transmissible gastroenteritis virus, porcine epidemic diarrhea virus, *Lactobacillus casei*, antibiotic-free selection, immunogenicity

## Abstract

Porcine transmissible gastroenteritis virus (TGEV) and porcine epidemic diarrhea virus (PEDV) are the causative agents of highly fatal acute diarrhea in pigs, resulting in enormous losses in the pig industry worldwide. To develop an effective bivalent oral vaccine against TGEV and PEDV infection, the D antigenic site of the TGEV spike (S) protein and the major antigen site (core neutralizing epitope—COE) of the PEDV S protein were used as immunogens, and the enhanced green fluorescent protein (eGFP) gene was used as a reporter to construct genetically engineered *Lactobacillus casei* rLpPG^F^-T7g10-eGFP-6D-COE. The expression of proteins of interest by the recombinant *L. casei* was confirmed by confocal laser scanning microscopy and a Western blot assay, and the immunogenicity of rLpPG^F^-T7g10-eGFP-6D-COE in orally immunized mice was evaluated. The results showed that levels of anti-PEDV and anti-TGEV serum immunoglobulin G (IgG) and mucosal secreted immunoglobulin A (sIgA) antibodies obtained from the mice immunized with rLpPG^F^-T7g10-eGFP-6D-COE, as well as the proliferation levels of lymphocytes, were significantly higher than those in mice orally administered phosphate-buffered saline (PBS) or rLpPG-T7g10. Moreover, the serum IgG antibodies showed neutralizing effects against PEDV and TGEV. Our data suggest that the antibiotic resistance-free genetically engineered *L. casei* bivalent oral vaccine provides a safe and promising strategy for vaccine development against PEDV and TGEV.

## 1. Introduction

Porcine transmissible gastroenteritis virus (TGEV) and porcine epidemic diarrhea virus (PEDV), which belong to the genus *Coronavirus*, family Coronaviridae, and order Nidovirales, are the main pathogens that cause highly fatal acute diarrhea in newborn piglets. This is followed by vomiting, dehydration, and high mortality, resulting in enormous losses in the pig industry worldwide [1]. Currently, porcine transmissible gastroenteritis (TGE), first reported in the United States in 1933, has spread to numerous European, South American, and Asian countries [2,3,4]. Porcine epidemic diarrhea (PED), which has been sporadic or locally endemic over the past three decades, experienced a larger-scale outbreak in the United States in 2013. Herds vaccinated with the CV777-inactivated vaccine were also infected, resulting in tremendous losses to the swine industry [5,6,7]. Accumulating evidence indicates that this large-scale recurrence of PED was caused by highly virulent PEDV variants [8,9,10,11]. In addition, co-infection of TGEV and PEDV often causes higher morbidity and mortality in newborn piglets. Therefore, the development of a safe and highly efficient vaccine against TGE and PED would be of great importance.

It is now clear that the gastrointestinal mucosas are the primary sites of PEDV and TGEV infection [12,13] and that mucosal immunization is a promising way to prevent PEDV and TGEV infection. However, the existing live attenuated/inactivated vaccines administered parenterally cannot effectively induce mucosal immunity against PEDV and TGEV infection [14,15,16,17]. Although some vaccines for TGE and PED developed in China can effectively stimulate intestinal mucosal immunity through Houhai acupoint injection, this immunization procedure is more time-consuming and labor-intensive. Therefore, vaccines that can induce a mucosal immune response against PEDV and TGEV would be of great significance, in particular those demonstrated to be safe, inexpensive, easy to use, and effective. The spike (S) glycoproteins of TGEV and PEDV possess antigen epitopes, and previous reports have demonstrated that the D antigen site (amino acids 378–392) in the S protein of TGEV and the core neutralizing epitope (COE; amino acids 499–638) in the S protein of PEDV can elicit neutralizing antibodies against TGEV and PEDV infection, respectively. This suggests that these are promising candidate antigens for the development of a genetically engineered vaccine [12,18,19].

*Lactobacillus casei* (*L. casei*) is a potential delivery vehicle for oral vaccines, because it is a probiotic bacterium characterized by its safety and resistance to gastric acid and bile [20,21]. Previous reports have shown that recombinant *L. casei* live vaccine is able to colonize the murine intestines for five days [22] and the swine intestines for longer [23]. Moreover, we have previously constructed a recombinant *L. casei* live vaccine expressing the D antigen site of the TGEV S glycoprotein combined with muramyl dipeptide and tuftsin as adjuvants, suggesting the possibility of a promising oral vaccine against TGEV challenge [12]. However, plasmid-mediated antibiotic resistance is commonly used as a selective marker for genetically engineered bacteria [24,25,26]. This could result in potential biosafety issues due to the transfer of antibiotic resistance from genetically engineered bacteria to environmental pathogens. Enhanced green fluorescent protein (eGFP) is a luminescent jellyfish protein with the amino acid substitutions necessary to generate a strong fluorescence signal when excited by ultraviolet or blue light. It is widely used in biological research, including in studies of cell differentiation, gene tracking, and protein localization and operation in vivo [27,28], providing a potential candidate for a screening marker to replace antibiotic resistance.

In this study, a genetically engineered *L. casei* strain, rLpPG^F^-T7g10-eGFP-6D-COE, was constructed using the TGEV S protein D antigen site and PEDV S protein-neutralizing antigen epitope region COE as immunogens, *L*. *casei* 393 as an antigen delivery vehicle, and eGFP as a selective marker, combined with a constitutive expression plasmid, pPG-T7g10. The immunogenicity of this strain when orally administered in mice was evaluated, suggesting a potential approach for the prevention of TGEV and PEDV infection.

## 2. Materials and Methods

All applicable international and national guidelines for the care and use of animals were followed. Approval (2016NEFU-315, 13 April 2017) was obtained from the Institutional Committee of Northeast Agricultural University for the animal experiments.

### 2.1. Virus, Plasmid, Bacterium, and Cell Lines

TGEV strain TH98 and PEDV strain HLJ-2012 were isolated by our laboratory from PEDV/TGEV-positive samples collected from pig farms in which a severe outbreak of acute diarrhea had been reported in piglets [29,30]. *L. casei* ATCC 393 was kindly provided by Jos Seegers (NIZO Institute, Netherlands). African green monkey kidney cells (Vero cells; ATCC CCL-81) and swine testicle (ST) cells were purchased from the China Center for Culture Collection (Wuhan, China) and were cultured in Dulbecco’s modified Eagle medium (DMEM; Gibco, Gaithersburg, MD, USA) supplemented with 10% fetal bovine serum (FBS; Gibco) at 37 °C with 5% CO_2_. The details of all plasmids used in this study are listed in Table 1.

### 2.2. Construction of the eGFP-Marked Recombinant Lactobacillus Strain

**Figure 1:**
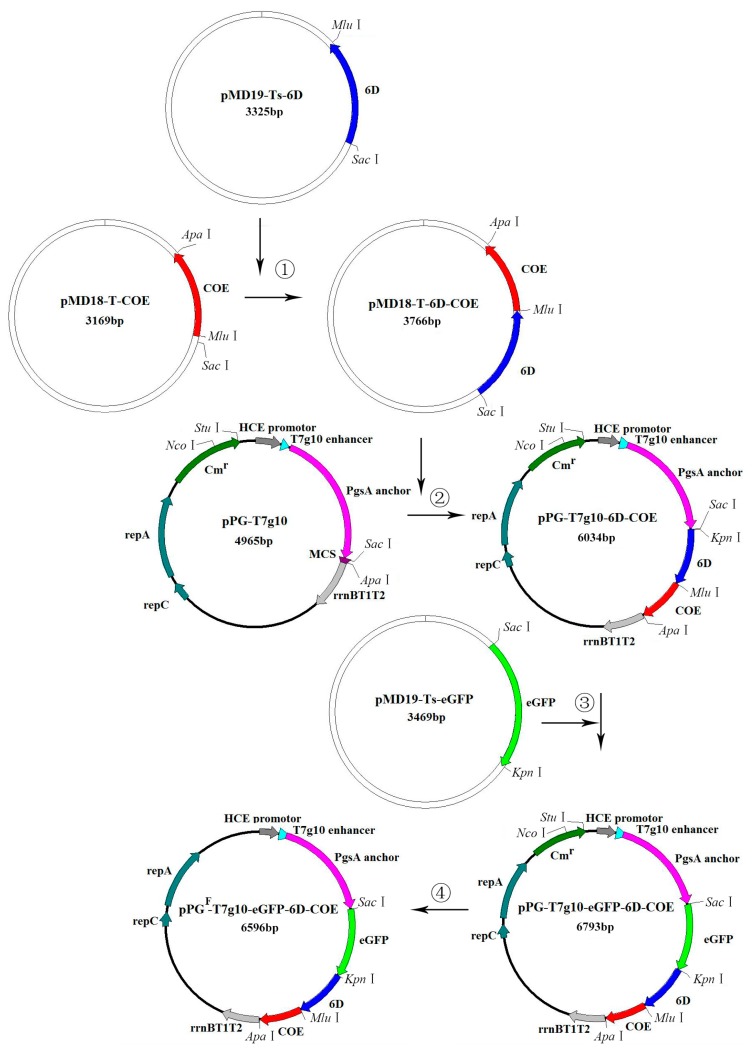
Schematic drawing of the construction of DNA plasmids. Plasmids pPG-T7g10-6D-COE and pPG^F^-T7g10-eGFP-6D-COE were generated according to the steps indicated by the arrows. ① The gene encoding 6D was inserted into plasmid pMD18-T-COE at *Sac*I and *Mlu*I sites, generating plasmid pMD18-T-6D-COE; ② fusion DNA fragment *6D-COE*, obtained from pMD18-T-6D-COE by *Sac*I and *Apa*I digestion, was inserted into the corresponding sites of plasmid pPG-T7g10, generating recombinant plasmid pPG-T7g10-6D-COE; ③ the gene encoding enhanced green fluorescent protein (eGFP) was inserted into pPG-T7g10-6D-COE at *Sac*I and *Kpn*I sites, generating recombinant plasmid pPG-T7g10-eGFP-6D-COE; ④ the chloramphenicol resistance (*Cm^r^*) gene as a selective marker in pPG-T7g10-eGFP-6D-COE was deleted using restriction enzymes *Stu*I and *Nco*I, followed by blunt-end treatment and ligation, giving rise to recombinant plasmid pPG^F^-T7g10-eGFP-6D-COE.

The primers used for amplifying genes encoding 6D (a peptide of the the D antigenic site of the TGEV spike (S) protein was repeated six times), COE, and eGFP are listed in Table 2. The linker sequence (GGGGS)3 was added in primers Fs2 and Re. A schematic diagram of the DNA plasmid construction is shown in Figure 1. In brief, the gene encoding 6D was inserted into plasmid pMD18T-COE at *Sac*I and *Mlu*I sites, generating plasmid pMD18T-6D-COE; then, the fusion DNA fragment *6D-COE*, obtained from pMD18T-6D-COE by *Sac*I and *Apa*I digestion, was inserted into the corresponding sites of plasmid pPG-T7g10, generating recombinant plasmid pPG-T7g10-6D-COE. Next, the gene encoding eGFP was inserted into pPG-T7g10-6D-COE at *Sac*I and *Kpn*I sites, generating recombinant plasmid pPG-T7g10-eGFP-6D-COE; finally, a chloramphenicol resistance (*Cm^r^*) gene selective marker in pPG-T7g10-eGFP-6D-COE was deleted using restriction enzymes *Stu*I and *Nco*I, followed by blunt-end treatment and ligation, giving rise to recombinant plasmid pPG^F^-T7g10-eGFP-6D-COE. All recombinant plasmids were identified by restriction enzyme digestion and sequencing.

For construction of the recombinant *Lactobacillus* strain, *L. casei* 393 competent cells were prepared according to a method previously described [34], followed by electroporation. Briefly, 50 ng of recombinant plasmid pPG^F^-T7g10-eGFP-6D-COE was gently mixed with 200 μL of *L. casei* 393 competent cells at 4 °C for 1 min; then, the mixture was transferred into a pre-cooled Gene Pulser (Bio-Rad, Hercules, CA, USA) disposable cuvette (inter-electrode distance of 0.2 cm) and subjected to a single electric pulse (1.5 V; 200 Ω; 25 μF) with a Gene Pulser (Bio-Rad). After growth at 37 °C for 8 h, the recombinant *Lactobacillus* strain with green fluorescence signal was collected through flow cytometry using a FACSCalibur (BD Biosciences, San Diego, CA, USA) at 488 nm and was grown on an de Man-Rogosa-Sharpe (MRS) plate at 37 °C for 36 h. This was followed by PCR confirmation and a chloramphenicol sensitivity assay, giving rise to recombinant strain rLpPG^F^-T7g10-eGFP-6D-COE. The hereditary stability of recombinant *L. casei* strains was detected, and rLpPG^F^-T7g10-eGFP-6D-COE was analyzed for stability by serially transferring the cultures after 24 h of incubation into MRS medium at 37 °C (1% inoculum; 50 generations). Plasmids were extracted from the cells, and PCR was used to confirm the presence of the gene *eGFP-6D-COE* in the plasmid using the primers Fe and Rs2.

### 2.3. Identification of Proteins Expressed by the Recombinant Strain

For analysis of the expression of proteins of interest by recombinant strains rLpPG-T7g10-6D-COE and rLpPG^F^-T7g10-eGFP-6D-COE, the bacterial strains were grown in basal MRS broth at 37 °C for 16 h (optical density of sample measured at a wavelength of 600 nm (OD_600_) ≈ 3.0) without shaking and were harvested by centrifugation at 10,000× *g* for 2 min. Cells were washed twice with sterile phosphate-buffered saline (PBS; pH 7.4) and lysed using a Mini-BeadBeater (BioSpec, Bartlesville, OK, USA). After centrifugation, the same quantity of total protein in the supernatants of each sample was isolated by sodium dodecyl sulfate 12% polyacrylamide gel electrophoresis (SDS-PAGE) and was transferred onto polyvinylidene fluoride membranes, followed by development with mouse anti-6D monoclonal antibody/rabbit anti-COE polyclonal antibody (diluted at 1:500) prepared in our lab, or mouse anti-eGFP monoclonal antibody (ZSGB-BIO, Beijing, China; diluted at 1:4000). Horseradish peroxidase (HRP)-conjugated goat anti-mouse/rabbit IgG antibody (Sigma, St. Louis, MO, USA) was utilized as a secondary antibody, diluted at 1:5000. Immunoblots were visualized with chemiluminescent substrate reagent (Pierce, Rockford, IL, USA) according to the manufacturer’s instructions.

Laser confocal microscopy was used to confirm the expression of fusion protein eGFP-6D-COE on the surface of rLpPG^F^-T7g10-eGFP-6D-COE using rLpPG-T7g10-6D-COE as a control. Briefly, recombinant strains were cultured in MRS medium at 37 °C for 16 h; then 1 mL of culture was collected by centrifugation at 5000× *g* for 5 min. The pellets were washed three times with PBS, re-suspended in 1 mL of PBS, and smeared on a microscope slide. Images were viewed by laser confocal microscopy (Zeiss, Oberkochen, Germany).

### 2.4. Immunizations

In order to evaluate the immunogenicity of recombinant strain rLpPG^F^-T7g10-eGFP-6D-COE used as an oral vaccine, 5-week-old female specific pathogen-free (SPF) BALB/c mice (derived from Mus Musculus) (*n* = 60) were obtained from Liaoning Changsheng Biotechnology Co., Ltd. (Liaoning, China) and kept under SPF conditions for one week with free access to a standard chow diet and water, in accordance with institutional guidelines. Prior to oral administration, the recombinant *Lactobacillus* strains were cultured for 16 h in MRS medium without shaking, washed with sterile PBS, and re-suspended in PBS at a concentration of 10^10^ CFU mL^−1^. SPF BALB/c mice were randomly divided into four groups (15 mice per group): PBS, rLpPG-T7g10, rLpPG-T7g10-6D-COE, and rLpPG^F^-T7g10-eGFP-6D-COE. The immunization dosages are shown in Table 3. The mice were immunized once a day for 3 consecutive days and boosted twice at 2 week intervals. After immunization, serum samples were collected from the immunized mice on days 0, 7, 14, 21, 28 and 35, and were stored at −20 °C until they were required for use. Mucosal lavage samples were obtained from the vaginas of the mice by washing with 200 μL of sterile PBS (pH 7.4) and were stored at −20 °C until analysis. In addition, fecal samples were collected and treated according to a method previously described [35]. Briefly, a 0.1 g fecal pellet was suspended in 400 μL of PBS containing 1 mmoL L^−1^ phenylmethylsulfonyl fluoride (Sigma) and 1% bovine serum albumin (BSA) and was then incubated at 4 °C for 16 h. After centrifugation, the supernatants were stored at −20 °C until use.

### 2.5. ELISA Assay

To detect TGEV- and PEDV-specific antibodies in the collected samples, polystyrene microtiter plates were coated with purified TGEV/PEDV for 12 h at 4 °C, using cultured ST/Vero cells as a negative antigen control. After blocking with 5% skim milk at 37 °C for 2 h and washing three times with PBS-0.1% Tween 20 (PBST), serum and mucus samples serially diluted in PBS-1% BSA were added to wells in triplicate, and then plates were incubated for 1 h at 37 °C. After washing with PBST, HRP-conjugated goat anti-mouse IgG or IgA antibody (Invitrogen, Carlsbad, CA, USA) was added to each well (1:5000) and incubated for an additional 1 h at 37 °C. The substrate o-phenylenediamine dihydrochloride (Sigma) was used for color development, and the absorbance was measured at 490 nm.

### 2.6. TGEV and PEDV Neutralization by Mouse Immune Sera

The neutralizing capacities of serum antibodies obtained from the mice immunized with rLpPG^F^-T7g10-eGFP-6D-COE were determined. Briefly, the 50% tissue culture infective dose (TCID_50_) values of TGEV and PEDV were detected by the Reed–Muench method. Serum antibodies collected from the vaccinated mice on day 35 post-immunization were diluted at 1:10–1:320 (in a total of 50 μL), mixed with an equal volume of PEDV or TGEV (100 TCID_50_ per 100 μL) and incubated at 37 °C for 1 h. Then, the treated viruses were added to a confluent monolayer of Vero and ST cells cultured in 24-well plates. The cells were overlaid with 1% methylcellulose, and the plates were incubated at 37 °C in a 5% CO_2_ atmosphere and examined daily for five days for TGEV- and PEDV-specific cytopathic effects (CPE).

### 2.7. Antigen-Induced Proliferation of Lymphocytes In Vitro

On day 35 post-immunization, splenocytes from three mice from each group were prepared for a lymphocyte proliferation assay as previously described [34]. Briefly, 100 μL of splenocytes (5 × 10^7^ cells mL^−1^) was suspended in Roswell Park Memorial Institute (RPMI) 1640 medium containing 10% fetal calf serum and then transferred to a 96-well flat-bottom plate; the cells were re-stimulated for 72 h with 1.0, 5.0 or 25 μg mL^−1^ TGEV-6D protein and PEDV-COE protein (produced by *Escherichia coli* prepared in our lab), using 5.0 μg mL^−1^ concanavalin A (ConA) and culture medium as positive and negative controls, respectively. The plates were supplemented with 10 μL of 3-(4,5-dimethylthylthiazol-2-yl)-2,5-diphenyltetrazoliumbromide (MTT) per well and incubated for an additional 4 h, and then proliferation was measured using OD_490_ values. The experiment was carried out in triplicate, and the lymphocyte proliferation index in the spleen (SI) was calculated as the mean reading of triplicate antigen stimulation wells divided by the mean reading of triplicate negative control wells.

### 2.8. Statistical Analysis

Data are shown as the means ± standard errors of three replicates per test in a single experiment repeated three times. Tukey’s multiple comparison tests were used to analyze differences among the groups. A *p-*value of <0.05 was considered statistically significant, and *p* < 0.01 was considered highly significant.

## 3. Results

### 3.1. Construction of eGFP-Marked Recombinant Lactobacillus Strain

**Figure 2:**
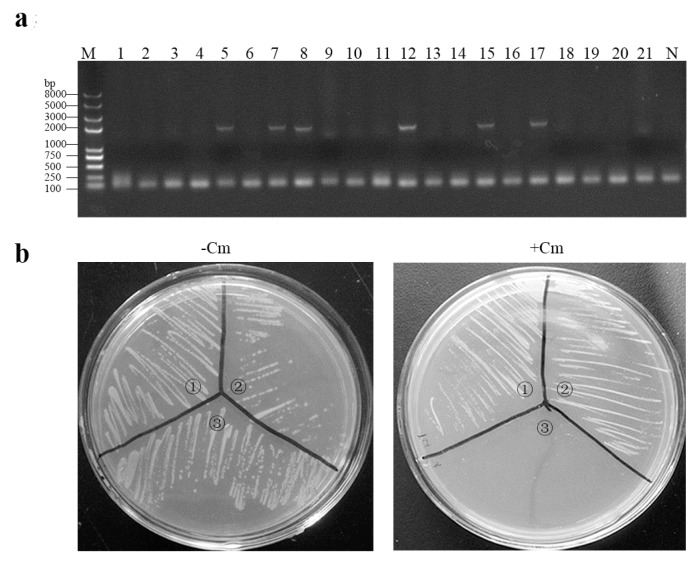
The identification of recombinant *Lactobacillus* strain rLpPG^F^-T7g10-eGFP-6D-COE without antibiotic resistance. (**a**) Recombinant strains with green fluorescence signal were collected, spread on an de Man-Rogosa-Sharpe (MRS) plate, and confirmed by colony PCR (Fe and Rs2 as primers). Lane M: Trans2K plus II DNA marker; lanes 1–21: colonies; lane N: negative control; (**b**) the results of chloramphenicol sensitivity assay. ① rLpPG-T7g10-6D-COE; ② rLpPG-T7g10-6D-COE; ③ rLpPG^F^-T7g10-eGFP-6D-COE.

Following flow cytometry screening and growth on antibiotic-free MRS plates, eGFP-marked recombinant *Lactobacillus* strain rLpPG^F^-T7g10-eGFP-6D-COE was obtained and identified with colony PCR (Figure 2a). As shown in Figure 2b, the strain rLpPG^F^-T7g10-eGFP-6D-COE could not grow on MRS agar medium in the presence of chloramphenicol. The result of the hereditary stability of recombinant *L. casei* strains showed that rLpPG^F^-T7g10-eGFP-6D-COE is stable for more than 50 generations.

### 3.2. Expression of Proteins of Interest by Recombinant Lactobacillus Strain

**Figure 3:**
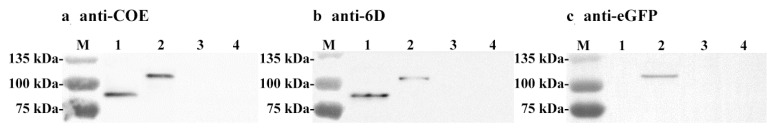
Western blot of recombinant *Lactobacillus* strain. Expression of poly-γ-glutamate synthase A (PgsA) fusion proteins by recombinant *Lactobacillus* was detected by Western blot with anti-COE (core neutralizing epitope) rabbit sera (**a**), anti-6D mouse sera (**b**), and anti-eGFP (enhanced green fluorescent protein) mouse antibodies (**c**). Lane 1: rLpPG-T7g10-6D-COE; lane 2: rLpPG^F^-T7g10-eGFP-6D-COE; lane 3: rLpPG-T7g10; lane 4: *L. casei.*

**Figure 4:**
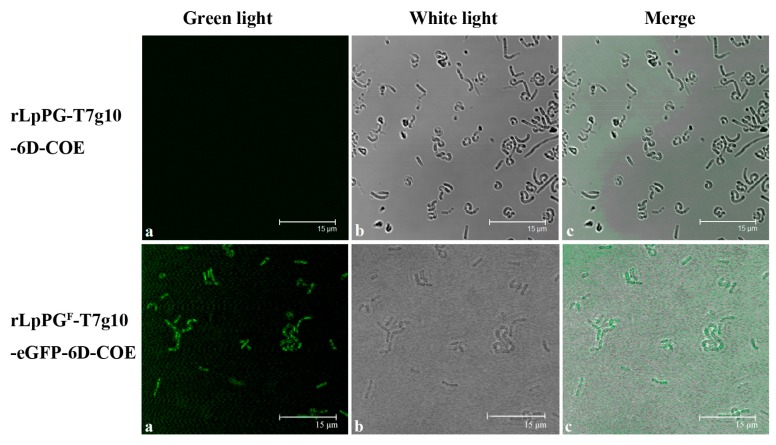
Expression of the PgsA-eGFP-6D-COE fusion protein on the cell surface. The localization of the PgsA-eGFP-6D-COE fusion protein in rLpPG^F^-T7g10-eGFP-6D-COE was detected by laser confocal microscopy. Bars are 15 μm for rLpPG^F^-T7g10-eGFP-6D-COE and rLpPG-T7g10-6D-COE. Recombinant strains were observed by green light (**a**) and white light (**b**) respectively. Panel (**c**) is a merged image of panel (**a**,**b**).

For confirming the expression of the proteins of interest by the recombinant *Lactobacillus* strain constructed in this study, the cell lysates of recombinant strains rLpPG-T7g10-6D-COE and rLpPG^F^-T7g10-eGFP-6D-COE were analyzed by a Western blot assay. As shown in Figure 3, the expected immunoblot bands of fusion protein pgsA-6D-COE of 80 kDa expressed by rLpPG-T7g10-6D-COE (Figure 3a,b) and the fusion protein pgsA-eGFP-6D-COE of 110 kDa expressed by rLpPG^F^-T7g10-eGFP-6D-COE were observed (Figure 3a–c), but these proteins were not expressed in rLpPG-T7g10 or *L. casei*. Moreover, green fluorescence could be observed by laser confocal microscopy on the surface of rLpPG^F^-T7g10-eGFP-6D-COE but not on rLpPG-T7g10-6D-COE (Figure 4), indicating that eGFP could successfully be used as a selective marker.

### 3.3. Immune Responses in Mice Induced by Recombinant Lactobacillus Strain

**Figure 5:**
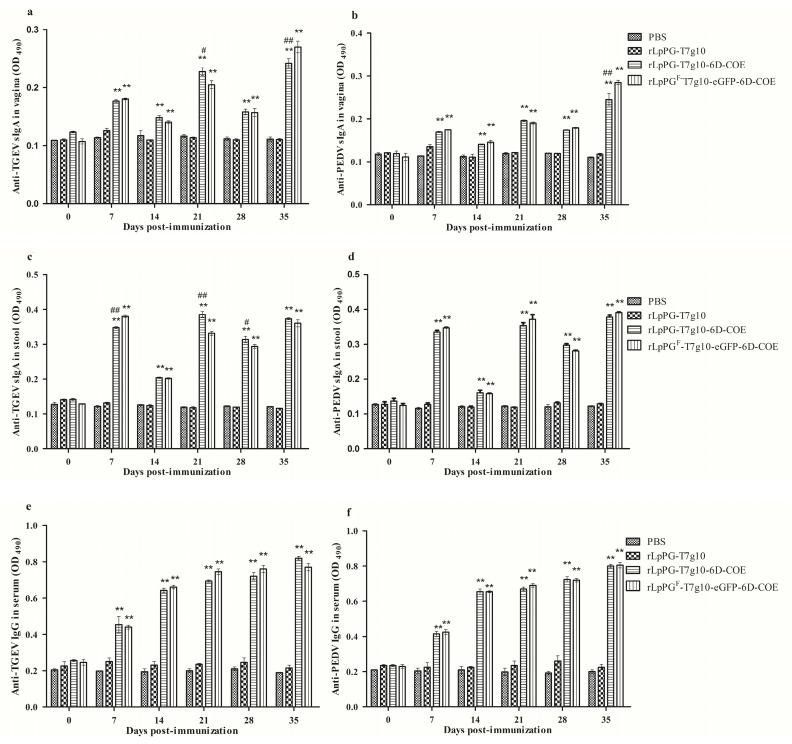
Levels of secreted immunoglobulin A (sIgA) and immunoglobulin G (IgG) in immunized mice. Transmissible gastroenteritis virus/porcine epidemic diarrhea virus (TGEV/PEDV)-specific sIgA antibody levels in the vagina (**a**,**b**) and the feces (**c**,**d**) post-immunization. Anti-TGEV IgG antibodies (**e**) and anti-PEDV-specific IgG antibodies (**f**) in immunized mice were detected at different time points. Bars represent the mean ± standard error of each group. * *p* < 0.05, ** *p* < 0.01 vs. phosphate-buffered saline (PBS) and vector control groups; # *p* < 0.05, ## *p* < 0.01 vs. rLpPG^F^-T7g10-eGFP-6D-COE group.

Anti-TGEV/PEDV-specific secreted IgA (sIgA) and IgG antibodies were assessed to evaluate the ability of rLpPG-T7g10-6D-COE and rLpPG^F^-T7g10-eGFP-6D-COE to induce mucosal and systemic immune responses using BALB/c mice as a model. As shown in Figure 5, anti-TGEV/PEDV-specific antibodies were detected at high levels 7 days post-immunization and were significantly increased after the booster immunization, peaking at 35 days post-immunization. The levels of anti-TGEV/PEDV-specific mucosal sIgA in mouse feces and vaginas, as well as the levels of anti-TGEV/PEDV-specific serum IgG antibody in mice orally immunized with rLpPG-T7g10-6D-COE/rLpPG^F^-T7g10-eGFP-6D-COE, were significantly higher (*p* < 0.01) than those in the PBS or rLpPG-T7g10 groups. In addition, the levels of anti-TGEV/PEDV sIgA in the vaginas of mice orally immunized with rLpPG^F^-T7g10-eGFP-6D-COE 35 days post-immunization were significantly higher (*p* < 0.01) than those in the rLpPG-T7g10-6D-COE group. There was no statistical difference (*p* > 0.05) observed in the PBS or rLpPG-T7g10 groups before and after immunization.

### 3.4. TGEV and PEDV Neutralization

**Figure 6:**
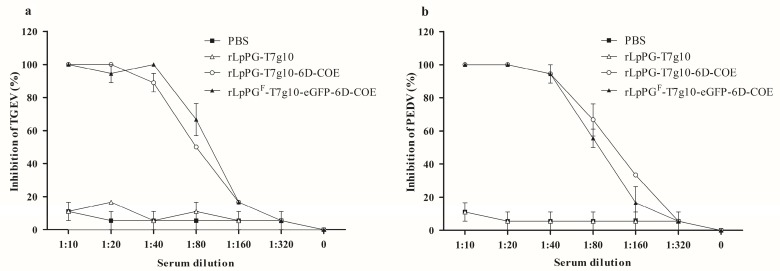
Neutralizing capacities of serum antibodies in immunized mice. Anti-PEDV (porcine epidemic diarrhea virus) (**a**), and anti-TGEV (transmissible gastroenteritis virus) (**b**) neutralizing antibodies in immunized mice were detected by plaque reduction assay with different serum dilutions from blood samples taken at 35 days post-immunization.

The neutralizing capacities of the serum antibodies induced in mice orally immunized with recombinant strains against TGEV (Figure 6a) and PEDV (Figure 6b) showed inhibitory activity against viral infection. The anti-TGEV neutralizing antibody titers were 10^−2^ (1:100) and 10^−1.933^ (1:85.7) and the anti-PEDV neutralizing antibody titers were 10^−1.967^ (1:92.7) and 10^−2.05^ (1:112.2) in mice immunized with rLpPG^F^-T7g10-eGFP-6D-COE and rLpPG-T7g10-6D-COE, respectively. These were significantly different from those obtained in the rLpPGF-T7g10 and PBS groups (*p* < 0.05).

### 3.5. Lymphocyte Proliferation

**Figure 7:**
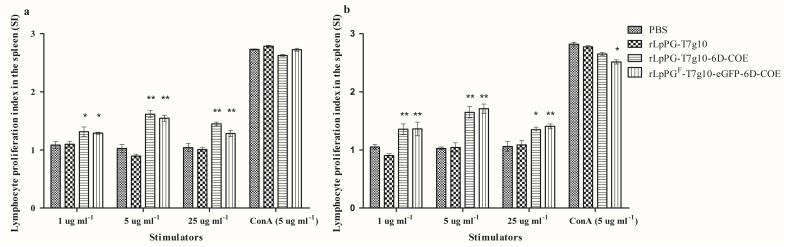
Proliferation of T lymphocytes from immunized mice. Lymphocyte proliferation was determined by 3-(4,5-dimethylthylthiazol-2-yl)-2,5-diphenyltetrazoliumbromide (MTT) assay with recombinant core neutralizing epitope (COE) protein (**a**) or D protein (**b**) as stimulating agents and concanavalin A (ConA) as a positive control. * *p* < 0.05, ** *p* < 0.01 vs. phosphate-buffered saline (PBS) and vector control groups.

The proliferation of spleen lymphocytes upon stimulation with purified COE or 6D proteins was analyzed by an MTT assay. The results showed that the proliferation levels of spleen lymphocytes from mice orally immunized with rLpPG-T7g10-6D-COE and rLpPG^F^-T7g10-eGFP-6D-COE were significantly higher than those of spleen lymphocytes from mice in the rLpPGF-T7g10 and PBS control groups (*p* < 0.01). There was no significant difference (*p* > 0.05) observed between the rLpPG-T7g10-6D-COE and rLpPG^F^-T7g10-eGFP-6D-COE groups (Figure 7).

## 4. Discussion

Over the past decades, large-scale outbreaks of diarrhea in swine caused by PEDV and TGEV have occurred in America, Europe, and Asia, resulting in considerable economic losses to the pig industry; in particular, PEDV infection has been responsible for many of these outbreaks [7,8]. The PEDV strain HLJ-2012 used in this study was isolated from an outbreak of acute diarrhea in piglets in Hegang, Heilongjiang Province, China [29], and phylogenetic analysis based on the *S* gene revealed that HLJ-2012 shares high homology with U.S. PEDV strains. Both belong to the GIIa subgroup, which represents epidemic and pandemic field strains [29,36]. Occasionally, changes in the antigenicity of PEDV due to amino acid mutations have resulted in vaccination failure [5,7]. The S protein neutralizing antigenic epitopes of PEDV have been reported to provide cross-protection against variant strains [9,29]. Therefore, we selected the COE of the PEDV S protein as an immunogen for developing an effective vaccine against PEDV infection.

PEDV and TGEV infections initially occur on mucosal surfaces, especially the intestinal mucosal epithelial surface. Therefore, mucosal vaccination is an effective strategy for preventing viral diarrheal diseases [12]. In this study, we used *L. casei* to deliver the TGEV S protein protective D antigenic site (repeated six times) and the PEDV S protein COE for developing an oral mucosal vaccine against PEDV and TGEV. Our results suggested that the genetically engineered *L*. *casei* strain rLpPG^F^-T7g10-eGFP-6D-COE can be used as a bivalent oral vaccine for PEDV and TGEV, eliciting mucosal and humoral immune responses against both TGEV and PEDV via oral immunization. This was evidenced by significantly higher levels of virus-neutralizing antibodies, anti-PEDV/TGEV serum IgG, and mucosal sIgA in mice orally immunized with rLpPG^F^-T7g10-eGFP-6D-COE, compared to the levels for the rLpPG-T7g10 or PBS groups. Moreover, the genetically engineered *L*. *casei* vaccine is safe and easy to administer, making it practical and convenient.

Mucosal immunity plays an important role in preventing viral diarrheal diseases, and sIgA antibodies from durable lactogenic immunity are a good way for piglets to obtain passive immunoprotection, indicating the importance of the sIgA antibody in the control of viral infection. At the same time, the level of sIgA can reflect the status of viral infection and the protective efficacy of vaccines [34]. In the present study, anti-TGEV/PEDV sIgA antibodies could be effectively induced at high levels in the feces and vaginas of mice orally treated with rLpPG^F^-T7g10-eGFP-6D-COE. IgA is reported to peak at 6 weeks and decline at 8 weeks in piglets [26]; thus, if durable immunity was present at 35 days post-immunization, newborn pigs could obtain immune protection. Therefore, we assessed the levels of the sIgA antibody for 35 days post-immunization. Our data showed that the levels of sIgA in the feces of mice orally immunized with rLpPG^F^-T7g10-eGFP-6D-COE gradually increased and peaked at 7, 21 and 35 days post-immunization, indicating that a mucosal immune response can be effectively elicited by rLpPG^F^-T7g10-eGFP-6D-COE after oral immunization. Therefore, the developed vaccine provides a promising strategy for protecting piglets from PEDV and TGEV infection via oral immunization.

Moreover, the neutralizing activity of an antibody is an important index used for evaluating the immunoprotective efficacy of a vaccine. In this study, high levels of serum antibody were elicited following oral immunization with rLpPG^F^-T7g10-eGFP-6D-COE. This could effectively neutralize the PEDV/TGEV infection, and higher antibody titers reflected higher neutralizing activities. As a comparison, the neutralizing antibody titer induced by the recombinant *L. casei* oral vaccine developed in this study was higher than that induced by a previously developed DNA vaccine [24]. Therefore, oral immunization with genetically engineered *L. casei* strain rLpPG^F^-T7g10-eGFP-6D-COE may provide effective protection for piglets against PEDV and TGEV infection.

In addition, our results showed that the eGFP-marked recombinant *Lactobacillus* oral vaccine rLpPG^F^-T7g10-eGFP-6D-COE (antibiotic-free selective marker vaccine) exhibited a similar immunogenicity to the antibiotic resistance marker vaccine rLpPG-T7g10-6D-COE, as the levels of antibodies induced by the rLpPG^F^-T7g10-eGFP-6D-COE and rLpPG-T7g10-6D-COE vaccines were not significantly different (*p* > 0.05). Notably, the use of eGFP as a selective marker in rLpPG^F^-T7g10-eGFP-6D-COE would avoid the main disadvantage of traditional plasmid expression systems by eliminating the use of antibiotic resistance genes as selective markers for genetically engineered bacteria [18,24,25]. Moreover, rLpPG^F^-T7g10-eGFP-6D-COE was constructed with a constitutive expression plasmid developed by our lab, exhibiting a significant advantage to inducible gene expression systems that require the use of an inductive agent. Additionally, a *pgsA*-derived anchoring matrix from *Bacillus subtilis* [37,38] was used to express the fusion proteins, which were displayed on the bacterial surface, eliciting good immunogenicity. The improved plasmid expression system used in this study therefore provides a powerful tool for the development of recombinant *Lactobacillus* oral vaccines.

## 5. Conclusions

In conclusion, an eGFP-marked recombinant *Lactobacillus* oral vaccine, rLpPG^F^-T7g10-eGFP-6D-COE, was constructed in this study and provides a promising strategy for the development of a bivalent oral vaccine against TGEV and PEDV infection. Further investigations are underway to evaluate the immunogenicity of this vaccine following its oral administration in piglets and to optimize the immunization procedures for effective control of TGE and PED.

## Figures and Tables

**Table 1:** Details of plasmids used in this study.

Plasmids	Relevant Characteristics	Description/Reference
pMD18-T-6D	As a template for PCR to amplify DNA fragment *6D*	Liu et al. 2011 [31]
peGFP-N1	As a template for PCR to amplify DNA fragment *eGFP*	Wang et al. 2016 [32]
pMD18-T-COE	3169 bp; *Amp^r^*; core neutralizing epitope (*COE*) of PEDV strain HLJ-2012	This study
pMD19-Ts	2692 bp; *Amp^r^*	Takara (DaLian, China)
pMD19-Ts-6D	3325 bp; *Amp^r^*; the amplified DNA fragment *6D* was inserted into pMD19-Ts	This study
pMD19-Ts-eGFP	3469 bp; *Amp^r^;* the amplified DNA fragment *eGFP* was inserted into pMD19-Ts	This study
pMD18-T-6D-COE	3766 bp; *Amp^r^*; *6D* was inserted into pMD18-T-COE	This study
pPG-T7g10	4965 bp; *Cm^r^*; HCE promoter of Geobacillus toebii D-AAT; poly-γ-glutamate synthase A (PgsA) anchor; constitutive expression plasmid	Song et al., 2014 [33]
pPG-T7g10-6D-COE	6034 bp; *Cm^r^*; *6D-COE* was inserted into pPG-T7g10	This study
pPG-T7g10-eGFP-6D-COE	6793 bp; *Cm^r^*; *eGFP* was inserted into pPG-T7g10-6D-COE	This study
pPG^F^-T7g10-eGFP-6D-COE	6596 bp; *Cm^r^* gene of pPG-T7g10-eGFP-6D-COE was deleted	This study

*Amp^r^*: ampicillin resistance; *Cm^r^*: chloramphenicol resistance.

**Table 2:** Details of primers used in this study.

Gene	ID	Primer Sequences	Length(bp)	Accession No./Reference
6D	Fs1	5′-GAGCTCGCAGGTACCAGATCTTGTT-3′	612	Liu et al., 2011 [31]
	Rs1	5′-ACGCGTGAGTCTAGAGGATCCGCCAC-3′		
COE	Fs2	5′-ACGCGT**GGTGGAGGAGGTTCAGGCGGAGGTGGCTCTGGCGGTGGCGGATCG**GTTACTTTGCCATCATTT-3′	420	JX512907
	Rs2	5′-GGGCCCAACGTCCGTGACACCTTC-3′		
eGFP	Fe	5′-CGAGCTCATGGTGAGCAAGGGCGA-3′	720	U55762.1
	Re	5′-GGGGTACC**CGATCCGCCACCGCCAGAGCCACCTCCGCCTGAACCGCCTCCACC**CTTGTACAGCTCGTCCATGC-3′		

Restriction enzyme recognition sites used for cloning are shown with underline. The linker (GGGGS)3 bases are shown in bold. 6D: a peptide of the D antigenic site of the porcine transmissible gastroenteritis virus spike (S) protein was repeated six times, COE: core neutralizing epitope in the S protein of porcine epidemic diarrhea virus, eGFP: enhanced green fluorescent protein.

**Table 3:** Groups and immunization dosages.

Group	No. of Mice	Dose/Treatment	No. of Treatments
Phosphate-buffered saline (PBS)	15	200 μL of PBS	3
rLpPG-T7g10	15	200 μL of 10^10^ CFU mL^−1^	3
rLpPG-T7g10-6D-COE	15	200 μL of 10^10^ CFU mL^−1^	3
rLpPG^F^-T7g10-eGFP-6D-COE	15	200 μL of 10^10^ CFU mL^−1^	3

CFU mL^−1^: colony-forming unit per mL of recombinant bacteria.
